# Supplementary material for: SALL4: An Intriguing Therapeutic Target in Cancer Treatment
Source: Cells. 2022 Aug 20;11(16):2601. doi: 10.3390/cells11162601 (PMC9406946; doi:10.3390/cells11162601)
Supplement: Supplementary file 1 [file cells-11-02601-s001.zip › Supplementary Data S1.pdf]

Supplementary Data S1:

The depmap cancer dependency data was exploited using the depmap R package.

#The needed packages for data analysis:

```
library("depmap")
library("ExperimentHub")
library("dplyr")
library("ggplot2")
library("stringr")
```

#The depmap datasets used in this study:

#gene expression data

```
TPM <- depmap_TPM()
```

#CRISPR-Cas9 gene knock out data

```
crispr <- depmap_crispr()
```

RNA-seq gene expression data

```
SALL4e::select(depmap_id, lineage) %>%
SALL4e::full_join(TPM, by = "depmap_id") %>%
sall4e::filter(gene_name == "SALL4") %>%
ggplot(aes(x = lineage, y = rna_expression, fill = lineage)) +
geom_boxplot(outlier.alpha = 0.1) +
theme(axis.text.x = element_text(angle = 45, hjust = 1)) +
theme(legend.position = "none")
```

#Calculation of mean CRISPR dependency

```
mean_crispr_dep <- crispr %>%
dplyr::select(gene_name, dependency) %>%
dplyr::filter(gene_name == "SALL4")

ggplot(aes(x = dependency)) + geom_histogram() +
geom_vline(xintercept = mean(mean_crispr_dep$dependency, na.rm =
TRUE),
linetype = "dotted", color = "red") +
geom_vline(xintercept = mean(crispr$dependency, na.rm = TRUE),
linetype = "dotted", color = "blue")
```

#CRISPR dependency score for SALL4 in different cell lineages

```
dplyr::select(depmap_id, lineage) %>%
dplyr::full_join(crispr, by = "depmap_id") %>%
dplyr::filter(gene_name == "SALL4")
```
